# Nutritional and Pharmacological Targeting of the Calcium-Sensing Receptor Influences Chemically Induced Colitis in Mice

**DOI:** 10.3390/nu11123072

**Published:** 2019-12-16

**Authors:** Taha Elajnaf, Luca Iamartino, Ildiko Mesteri, Christian Müller, Marcella Bassetto, Teresa Manhardt, Sabina Baumgartner-Parzer, Enikö Kallay, Martin Schepelmann

**Affiliations:** 1 Center of Pathophysiology Infectiology and Immunology, Medical University of Vienna, Pathophysiology and Allergy Research, Währinger Gürtel, 18-20, 1090 Vienna, Austria; taha.elajnaf@meduniwien.ac.at (T.E.); luca.iamartino@meduniwien.ac.at (L.I.); christian_muell_er@hotmail.com (C.M.); teresa.manhardt@meduniwien.ac.at (T.M.); martin.schepelmann@meduniwien.ac.at (M.S.); 2 Pathologie Überlingen, 88662 Überlingen, Germany; ildiko.mesteri@hotmail.com; 3 School of Pharmacy and Pharmaceutical Sciences, Cardiff University, King Edward VII Avenue, CF10 3NB Cardiff, UK; 4 Department of Chemistry, College of Science, Swansea University, SA2 8PP Swansea, UK; 5 Department of Internal Medicine III, Medical University of Vienna, 1090 Vienna, Austria

**Keywords:** IBD, colitis, inflammation, calcium, mucin

## Abstract

The calcium-sensing receptor (CaSR) is the main regulator of extracellular Ca^2+^ homeostasis. It has diverse functions in different tissues, including the intestines. Intestine-specific knockout of the CaSR renders mice more susceptible to dextran sulphate sodium (DSS)-induced colitis. To test our hypothesis that the CaSR reduces intestinal inflammation, we assessed the effects of nutritional and pharmacological agonists of the CaSR in a colitis model. We treated female Balb/C mice with dietary calcium and protein (nutritional agonists of the CaSR) or pharmacological CaSR modulators (the agonists cinacalcet and GSK3004774, and the antagonist NPS-2143; 10 mg/kg), then induced colitis with DSS. The high-protein diet had a strong pro-inflammatory effect—it shortened the colons (5.3 ± 0.1 cm vs. 6.1 ± 0.2 cm normal diet, *p* < 0.05), lowered mucin expression and upregulated pro-inflammatory cytokines, such as interferon-γ, (4.2-fold, *p* < 0.05) compared with the normal diet. Cinacalcet reduced mucin expression, which coincided with an increase in tumor necrosis factor-α (4.4-fold, *p* < 0.05) and IL-6 (4.9-fold, *p* < 0.05) in the plasma, compared with vehicle. The CaSR antagonist, NPS-2143, significantly reduced the cumulative inflammation score compared with the vehicle control (35.3 ± 19.1 vs. 21.9 ± 14.3 area under the curve, *p* < 0.05) and reduced infiltration of inflammatory cells. While dietary modulation of the CaSR had no beneficial effects, pharmacological inhibition of the CaSR may have the potential of a novel add-on therapy in the treatment of inflammatory bowel diseases.

## 1. Introduction

The calcium-sensing receptor (CaSR) is a dimeric G protein-coupled receptor (GPCR) that plays a central role in calcium homeostasis. The CaSR is expressed at highest levels in the parathyroid glands and kidneys, where it regulates parathyroid hormone release and calcium reabsorption [1,2]. What distinguishes the CaSR from other members of the GPCR family is its sensitivity to minute alterations in extracellular Ca^2+^ over the millimolar range, which enables it to function as the body’s calciostat [3]. Besides extracellular Ca^2+^, the CaSR responds to numerous ligands including divalent and trivalent cations, polyamines, amino acids, antibiotics and other external stimuli such as pH and ionic strength [4]. At the cellular level, the CaSR fine-tunes numerous signaling pathways downstream of G_i_, G_Q/11_, G_12/13_ and β-arrestin in a ligand- and tissue-dependent manner [5,6,7,8,9,10]. This is evident in the diversity of the physiological processes that the CaSR regulates, spanning calcitropic and non-calcitropic tissues, i.e., those not directly involved in calcium homeostasis. These include proliferation, differentiation, hormone secretion and excitability to name but a few [1]. There is increasing interest in the role of the CaSR in non-calcitropic tissues including the gastrointestinal tract (GI), where the CaSR has been suggested to function as a nutrient sensor and to regulate intestinal motility, fluid transport and inflammation [11,12,13,14,15]. Accordingly, the CaSR may potentially be a therapeutic target for toxin-mediated diarrhea and intestinal inflammation.

There exists a reciprocal relationship between the CaSR and inflammation. Inflammatory cytokines (tumor necrosis factor-α (TNF-α), interleukin-1β (IL-1β) and IL-6) regulate the transcription of the CaSR, while the CaSR itself broadly regulates inflammation in a tissue-dependent manner (see reference [11] for a full review). For example, in the lung, the CaSR has pro-inflammatory effects and thus offers a therapeutic potential for CaSR antagonists to treat asthma [16]. Conversely, previous studies suggested a protective role for the CaSR in intestinal inflammation, which highlights the CaSR as a promising target for inflammatory bowel disease (IBD) treatment [17,18,19]. IBD encompasses Crohn’s disease, which affects the entire gastrointestinal tract, and ulcerative colitis, which only affects the colon. There is no cure for either disease. Animal models for IBD, such as the dextran sulphate sodium (DSS)-induced colitis model, are widely used in preclinical drug development [20]. Intake of DSS in drinking water either acutely or chronically results in symptoms that are reminiscent of colitis.

Initial findings linking the CaSR to intestinal inflammation come from a study on DSS-induced colitis by Cheng et al., where knockout of the CaSR in intestinal epithelia increased susceptibility to DSS [17]. Loss of the CaSR in intestinal epithelia compromised the epithelial barrier, thus leading to infiltration by pathogens and immune cells and an imbalance in gut immunity, favoring inflammation [17]. It was also reported that high dietary calcium intake reduces intestinal inflammation and the associated risk for colorectal cancer [21,22]. The luminal CaSR along the GI tract is available to modulation by a plethora of ligands including divalent cations, amino acids, polyamines and glutamyl dipeptides as products of food intake and digestion. Therefore, the CaSR might represent a druggable target that is favorable for nutraceutical supplementation, as well as pharmaceutical intervention for treating IBD. Two independent studies by Zhang et al. demonstrated that amino acids and glutamyl dipeptides alleviate DSS-induced colitis symptoms in vivo and inhibit TNF-α-induced pro-inflammatory cytokine production in vitro [19,23]. More recently, Liu et al. also suggested that CaSR activation by aromatic amino acids mitigates intestinal inflammation in lipopolysaccharide-challenged piglets [24]. Despite the evidence presented in these studies, they have two major limitations. First, CaSR dependence is not adequately demonstrated, particularly in vitro where the cell lines used are reported to have little or no CaSR expression [25]. Second, although the ligands that were used are known agonists or allosteric modulators of the CaSR, they are not highly selective. Furthermore, to our knowledge, there are no reports on the effects of positive allosteric modulators (calcimimetics) such as, cinacalcet or NPS-R568, on intestinal inflammation.

To address these limitations, we have chosen two approaches to study the putative involvement of the CaSR in intestinal inflammation. First, we have used diets containing different levels of the main CaSR ligand, Ca^2+^, in addition to a high protein diet which is a major source of amino acids and peptides. Second, we used highly selective pharmacological CaSR ligands to target the luminal CaSR in the intestines and hypothesized that modulating the CaSR will influence DSS-induced colitis in mice. For a combined local and systemic effect, we used the clinically available calcimimetic cinacalcet (Sensipar/Mimpara). For a more localized effect and to achieve high drug concentrations in the intestines, we used - for the first time - a gut-restricted calcimimetic (GSK3004744) [26]. We also used the calcilytic NPS-2413 to inhibit the CaSR. We found that dietary protein exacerbated acute DSS-induced colitis. Moreover, we showed for the first time that cinacalcet had pro-inflammatory effects highlighted by a reduction in mucin and an increase in pro-inflammatory cytokines in the plasma. On the contrary, NPS-2143 alleviated clinical symptoms of colitis. Our study uncovered previously unknown effects of allosteric modulators of the CaSR on acute intestinal inflammation.

## 2. Materials and Methods

### 2.1. Mouse Handling and Maintenance

All animal experiments were approved by the Ethics Committee of the Medical University of Vienna and the Austrian Federal Ministry of Education, Science and Research (BMWFW-66.009/0401-WF/V/3b/) and carried out in accordance with the European Union Regulations on Care and Use of Laboratory Animals. Female BALB/c mice (8 weeks, 16−20 g; Charles River Laboratories, Inc., Sulzfeld/Grabfeld, Germany) were group housed on a 12 h−12 h light−dark cycle and allowed unrestricted access to the standard semi-synthetic diet AIN-93M (LASCRdiet™ LasVendi, Germany), which contained 0.5% calcium and 13% protein, or the experimental diets as indicated. All experimental diets were isocaloric and differed from the standard (control) diet only in calcium and protein content as follows: low calcium (0.05%), high calcium (1.5%) and high protein (26%). The amounts of dietary calcium and protein are listed in Table 1. The full composition of the diets is listed in Table S1.

### 2.2. Experimental Design and Induction of Colitis

**Figure 1:**
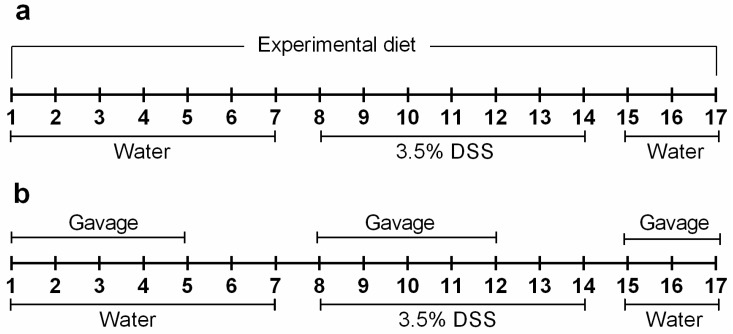
Schematic representation of the experimental design. Colitis was induced by dextran sulphate sodium (DSS) (3.5%) in drinking water for 7 days, followed by a 3-day resolution phase. (**a**) Mice received semi-synthetic diets (based on AIN-93M) differing in calcium and protein content as follows: normal calcium (0.5%), low calcium (0.05%), high calcium (1.5%) and high protein (26%), throughout the course of the experiment. (**b**) Mice received a daily dose (10 mg/Kg) of either vehicle (20% cyclodextrin) or calcium-sensing receptor (CaSR) allosteric modulators (NPS-2143, GSK3004774 or cinacalcet) by gavage; treatments started 7 days prior to DSS administration and continued until euthanasia.

The optimal concentration of DSS for induction of inflammation in BALB/c mice was determined in a pilot study. We tested 2.5, 3 and 3.5% DSS and determined that clear clinical symptoms were observed with 3.5% DSS. Thus, 3.5% DSS was given in drinking water ad libitum for 7 days followed by a short 3-day resolution phase. “No-DSS” control mice received normal drinking water. Two independent studies were carried out to determine the effects of dietary and pharmacological modulators of the CaSR on DSS colitis. In the diet study, the mice were randomly assigned into groups (10/group) and were fed ad libitum one of the following diets: normal calcium (0.5%), low calcium (0.05%), high calcium (1.5%) and high protein (26% protein). The mice were fed with the experimental diets for 3 weeks prior to DSS administration and continued until the end of the experiment (Figure 1A). For assessment of the effects of the allosteric modulators of the CaSR on DSS colitis, the mice were randomly assigned into groups (25/group) and received by oral gavage: cinacalcet (Tocris Bioscience, Bristol, UK), GSK3004744, or NPS-2143 (Tocris Bioscience, Bristol, UK) at 10 mg/Kg body weight, dissolved in 20% (2-hydroxypropyl)-β-cyclodextrin (Sigma Aldrich) in distilled water. Vehicle treated mice received 20% cyclodextrin only. Gavage, administered on weekdays, lasted for 2 weeks, beginning 1 week prior to the DSS treatment and until the day of euthanasia (Figure 1B). Animals were euthanized 3 days post-DSS or upon reaching the criteria for a humane endpoint (clinical score = 16, see next section). Blood was collected by heart puncture, centrifuged at 2000× *g* for 5 min and plasma was collected in tubes coated with lithium heparin and stored at −80 °C. Colons were removed from cecum to anus, flushed with PBS and their lengths and weights were measured. Colons were cut longitudinally; one half was rolled in a Swiss roll, fixed in 4% Roti-Histofix (Roth, Germany) and paraffin embedded for histological analysis. The remaining half was divided into right and left and snap frozen for protein extraction.

### 2.3. Clinical Assessment of Colitis

Mice were weighed and monitored weekly prior to DSS and gavage treatment. Throughout the DSS course, mice were monitored daily and scored for physical symptoms of colitis, under blinded conditions, according to the criteria by Chassing et al. [27] as follows. Physical appearance: 0 = normal, 1 = general lack of grooming, 2 = staring coat, ocular and nasal discharges, 4 = piloerection, hunched up. Body weight loss: 0 = normal 1 = 5–10%, 2 = 10–15%, 4 = > 15%. Behavior: 0 = normal, 1 = mild depression or exaggerated response, 2 = less mobile and alert, isolated, 4 = vocalization, self-mutilation, restless, violence, inactive, cold. Feces: 0 = normal, 1 = soft, positive fecal occult blood test, Haemoccult (Beckman Coulter), 2 = very soft with visible traces of blood, 4 = visible rectal bleeding. If no stool could be collected on one day, the average score of the adjacent former and latter days was used, or, in case of no possibility of further scoring, the score was continued to the end of the experiment.

### 2.4. Histology Scoring

Colon sections were processed as per standard histology protocol, formalin-fixed, embedded in paraffin and sliced into 4 µm sections. The sections were stained by hematoxylin and eosin and Images were acquired using TissueFAXS Hard and Software (TissueGnostics GmbH, Wien, Austria), using a 20x Objective (Neo-Fluar NA 0.5; Zeiss, Oberkochen, Germany). Scoring was carried out by an experienced pathologist under blinded conditions, and was based on evaluation of inflammation, ulceration, mucosal remodeling and number of lymph follicles.

### 2.5. Mucin Quantification

In order to determine the amount of mucin per epithelium, paraffin-embedded colon sections were stained by alcian blue for mucin and nuclear fast red and images were acquired using TissueFAXS Hard- and Software, using a 20× Objective (Neo-Fluar NA 0.5). Images were then downsized and converted to 8–bit images for analysis with Image J [28]. In order to quantify the total area of epithelium per section accurately, we excluded lymph follicles and staining artefacts using manual segmentation of the images. The muscularis layer was automatically segmented using the trainable Waikato environment for image analysis (WEKA) [29]. We then used color deconvolution [30] and thresholding to separate and quantify the mucin positive area per area of epithelium.

### 2.6. Cytokine Multiplex Assay

The 36-Plex Mouse ProcartaPlex Panel 1A (Thermo Fisher Scientific, Waltham, MA, USA) was used to quantify chemokines and cytokines from plasma and colon extracts as per the manufacturer’s instructions. Briefly, a minimum of 2 plasma samples from each group were pooled and 25 µL per pool was used in duplicates. Frozen right and left colon pieces were lysed in ProcartaPlex lysis buffer containing protease inhibitor (1:500) and phenylmethylsulfonyl fluoride (PMSF; 1 mM) in an automatic homogenizer (Precellys, Germany) at 6500 rpm for 10 seconds. Protein was then quantified by the BCA method, pooled with a minimum of 2 samples per pool and 250 µg per pool were loaded in duplicates.

### 2.7. Immunohistochemistry

Paraffin-embedded colon sections were incubated for 25 min at 60 °C, deparaffinized, and rehydrated. After washing with PBS (pH 7.2), sections were boiled in 0.05% citrate buffer for antigen retrieval, permeabilized with 0.2% Tween-20 in PBS for 20 min, and blocked with 5% goat serum in PBS for 30 min. Sections were simultaneously incubated with rabbit monoclonal anti-β-catenin antibody (Abcam, Cambrige, UK; 1:100) and rat monoclonal anti-Ki-67 antibody-eFluor 570 (eBioscience; 1:250) overnight at 4 °C, followed by extensive washing with PBS and incubation with secondary antibody: goat anti-rabbit-AlexaFluor 647 (1:2000). To detect immune cell infiltration, sections were incubated with rabbit anti-mouse cluster of differentiation T cell co-receptor 3 (CD3) (Abcam; 1:100) and mouse anti-mouse CD20 (Santa Cruz, CA, USA, 1:100) overnight at 4 °C, followed by extensive washing with PBS and incubation with secondary antibody: goat anti-rabbit-AlexaFluor 647 (1:2000). Rabbit and mouse IgG were used as negative controls. Nuclei were stained with DAPI (1:1000) for 10 min at room temperature and samples were mounted using Fluoromount-G. Whole section images were acquired with the automated TissueFAXS system (TissueGnostics GmbH, Wien, Austria).

### 2.8. Quantification of Immune Cell Infiltration

CD3^+^ and CD20^+^ cells were analyzed under blinded conditions in whole Swiss roll sections by TissueQuest Software (TissueGnostics GmbH, Wien, Austria) using nuclear segmentation based on nuclear DAPI staining, with nuclear and ring masks for the respective target protein to encompass the whole cell. Manual thresholding for positive cells was used for each target and then applied to all tissue sections. The analysis was performed separately for lymph nodes (marked manually) and extra-lymphatic tissue for CD3^+^ and CD20^+^ cells.

### 2.9. GSK3004774 Synthesis

GSK3004774 was synthesized according to literature procedures [26,31] and obtained as a 99% pure, 1:2 diastereomeric mixture of 1-(3-(4-((S)-3-(((R)-1-(naphthalen-1-yl)ethyl)amino)pyrrolidin-1- yl)benzamido)propyl)piperidine-4-carboxylic acid and 1-(3-(4-((R)-3-(((R)-1-(naphthalen-1-yl)ethyl) amino)pyrrolidin-1-yl)benzamido)propyl)piperidine-4-carboxylic acid).

### 2.10. Intracellular Ca^2+^ Measurements to Test the Activity of GSK3004774

Techniques using the ratiometric Ca^2+^ indicator fura-2 AM have been previously described [16]. In brief, HEK293 cells stably transfected with the human CaSR (HEK-CaSR; a kind gift from Professor Daniela Riccardi, Cardiff University) and grown on 13 mm glass coverslips coated in poly-D-lysine were loaded with Fura-2 AM (Thermo Fisher) for 2 hours at 37 °C. After washing, the cells were pre-incubated in extracellular solution (135 mM NaCl, 10 mM glucose, 5 mM KCl, 5 mM HEPES, 1.2 mM MgCl_2_) containing 0.2 mM Ca^2+^ and either vehicle (0.1% v/v DMSO) or 1 µM GSK3004774 for 10 min. Fluorescence of Fura-2 was then recorded at 340 and 380 nm using an inverted fluorescence microscope (IX71, Olympus. Shinjuku, Tokyo, Japan) at 2 second intervals. A rapid perfusion system allowed changing of the solutions. Fluorescence was recorded for 2 min at 0.2 mM Ca^2+^ (baseline), followed by 3 min at 3 mM Ca^2+^, followed again by 2 min at 2 mM Ca^2+^. Integrated response units (IRUs: trapezoidal integral of the timespan) were calculated of the baseline and the last two minutes of the 3 mM Ca^2+^ phase and the value for baseline was subtracted from the value for the 3 mM Ca^2+^ phase (ΔIRU). An increase in the intracellular Ca^2+^ response (increase in ΔIRU) in the GSK3004774 treated cells compared to vehicle treatment indicated a stronger activation of the CaSR and thus activity of the compound GSK3004774 as a calcimimetic (Figure S1a–d).

### 2.11. Statistical Analysis

Data are presented as the mean ± SEM or median ± interquartile range. Statistical analyses were carried out using GraphPad Prism version 7.0 (GraphPad, San Diego, CA, USA). Used statistical tests are indicated with the respective figures or tables.

## 3. Results

### 3.1. Clinical Symptoms of Colitis were Exacerbated by High Dietary Protein, but Improved by NPS-2143

During the acclimatization period and prior to colitis induction, we monitored body weight and determined that the different diets and the allosteric modulators had no influence on body weight (Figure S2a,b).

**Figure 2:**
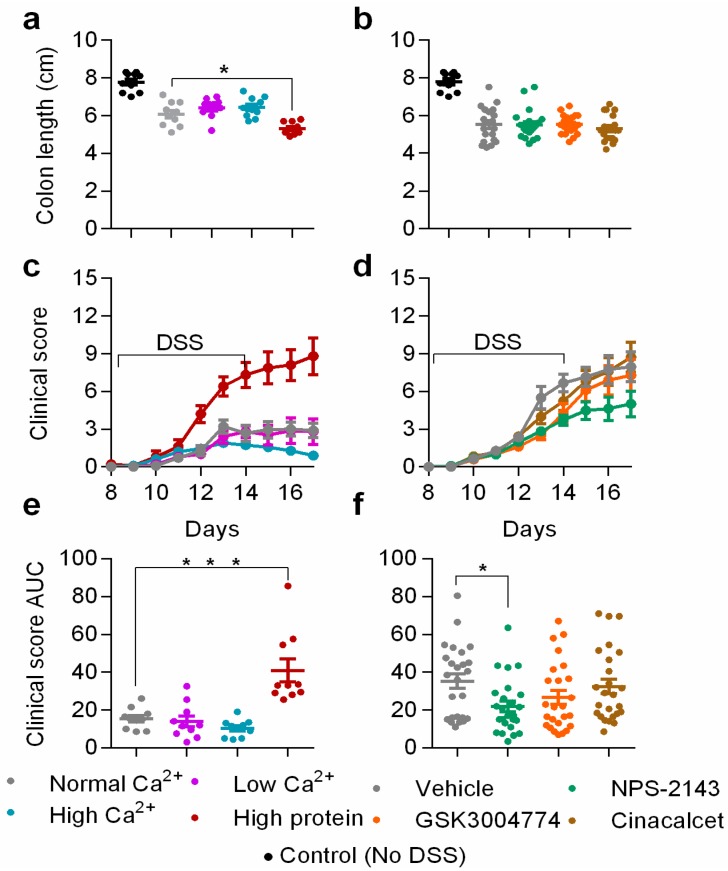
The effects of dietary protein, dietary calcium and CaSR allosteric modulators on colon length and clinical score of colitis. Colon lengths of colitic mice and no-DSS controls fed with different diets (**a**) or treated with CaSR allosteric modulators (**b**). Clinical score of colitic mice fed with different diets (**c**) or treated with CaSR allosteric modulators (**d**) Clinical score is based on (1) general appearance, (2) weight loss, (3) behavior (4) blood in stool. Area under the curve of the cumulative clinical score of colitic mice fed with different diets (**e**) or treated with CaSR allosteric modulators (**f**). Statistical significance was determined by one-way ANOVA or Kruskal–Wallis for ranked clinical scores. * *p* < 0.05, ** *p* < 0.01 and *** *p* < 0.001 were regarded as statistically significant. Data are presented as the mean ± SEM, *n* = 10 and 25 mice per group, for the diets and modulators experiments, respectively.

To determine the effects of dietary calcium and protein on colitis symptoms, we divided the mice into four groups and fed them the following diets: normal calcium (0.5%) and protein, low calcium, high calcium, and high protein (Table 1). DSS induced minor weight loss and a significant shortening of colons compared with the no-DSS control group (Figure 2a). The high protein diet caused a significant loss of weight compared with the normal diet group in colitic mice (Figure S2c). The colons of mice fed with the high protein diet were significantly shorter than those of the normal diet group (5.3 ± 0.11 and 6.1 ± 0.20 cm, *p* = 0.0102) (Figure 2a). The deleterious effects of the high protein diet were also reflected in the clinical score, which was highest in the high protein diet group (Figure 2c,e).

The high calcium diet had no effect on the clinical score during the induction phase. However, there was a small but not statistically significant reduction in the clinical score during the resolution phase (Figure 2c). The low calcium diet had no effect on clinical score compared with the normal diet group. One mouse died due to severe inflammation in the high protein diet group as shown in the survival curve (Figure S3a).

It is suggested that activation of the CaSR in the intestines attenuates DSS-induced colitis [18,19]. Therefore, we examined the effects of pharmacological CaSR modulators: two positive allosteric modulators, cinacalcet and GSK3004774, and the negative allosteric modulator NPS-2143. We tested the effectiveness of the allosteric modulators by measuring serum parathyroid hormone (PTH) levels. As expected, NPS-2143 increased, cinacalcet caused a statistically insignificant reduction, while GSK3004774 had no effect on PTH levels (Figure S4). All the DSS-treated groups which received either 20% cyclodextrin only as a vehicle or 10 mg/Kg of allosteric modulators experienced substantial weight loss (Figure S2d). This is likely a result of gavage which increased the inflammatory response, as reflected in the clinical score and pro-inflammatory cytokine expression in the plasma and in the left colon (Figure S5a–d). However, we observed no differences in the weight loss (Figure S2d) or colon length (Figure 2b) among the different treatment groups throughout the induction and resolution phases. Neither cinacalcet, nor GSK3004774 affected the clinical score. By contrast, NPS-2143 reduced the clinical score in the resolution phase (Figure 2d). We analyzed the cumulative score (area under the curve) for the clinical score (Figure 2f), which was significantly lower for the NPS-2143 group (21.9 ± 14.3) compared with the vehicle group (35.3 ± 19.1, *p* < 0.05). During colitis induction, several spontaneous fatalities were observed during the daily assessment and mice that reached the humane threshold (clinical score > 15) had to be euthanized. Interestingly, most mice survived in the group treated with NPS-2143 (88%), compared with vehicle- (64%), cinacalcet- (60%) and GSK3004774- (72%) treated groups (Figure S3b).

### 3.2. Histological Scores were Increased by High Dietary Protein, while Unaffected by Dietary Calcium and The Pharmacological Modulators of The CaSR

**Figure 3:**
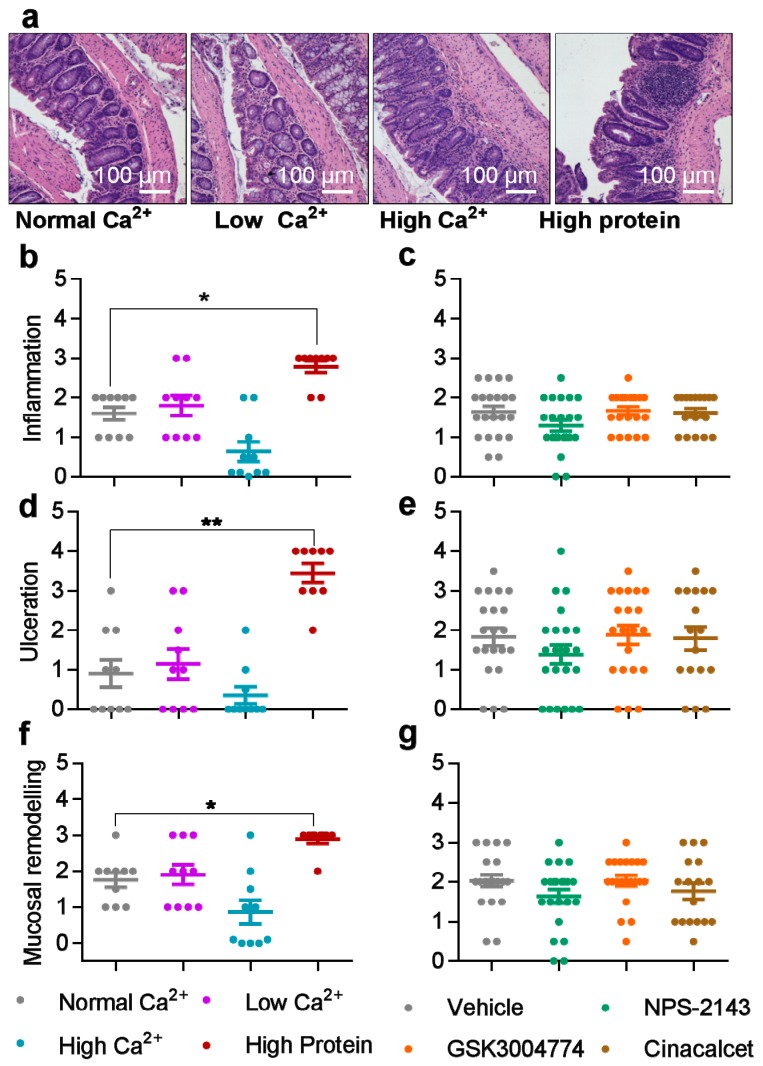
The effects of dietary calcium, dietary protein and allosteric modulators of the CaSR on histological scores of colitic mice. (**a**) Hematoxylin and eosin (H&E)staining of colon sections from colitic mice fed with different diets. Histological Inflammation score of colitic mice fed with different diets (**b**) or treated with CaSR allosteric modulators (**c**). Ulceration scores of colitic mice fed with different diets (**d**) or treated with CaSR allosteric modulators (**e**). Mucosal remodeling scores of colitic mice fed with different diets (**f**) or treated with CaSR allosteric modulators (**g**). Histological evaluation was carried out by an experienced pathologist under blinded conditions. Statistical significance was determined by Kruskal–Wallis test. * *p* < 0.05, ** *p* < 0.01 and *** *p* < 0.001 were regarded as statistically significant. Data are presented as median ± interquartile range, *n* = 10 and 25 mice per group, in the diets and modulators experiments, respectively.

DSS profoundly affected colon histology (Figure 3a). Histology score in the DSS model represents a key parameter in preclinical drug studies. The high protein diet caused increased tissue damage, which manifested as significantly higher inflammation, ulceration ad mucosal remodeling, compared with the normal/standard diet (Figure 3b,d,f). Neither the Ca^2+^ content (Figure 3b,d,f) nor any of the allosteric modulators (Figure 3c,e,g) affected the studied histological parameters. Moreover, Ki67 and β-catenin levels were not affected by any of the diets or the allosteric modulators of the CaSR (Figure S6a–d).

### 3.3. Mucin Was Reduced by High Dietary Protein and the Positive Allosteric Modulator Cinacalcet

**Figure 4:**
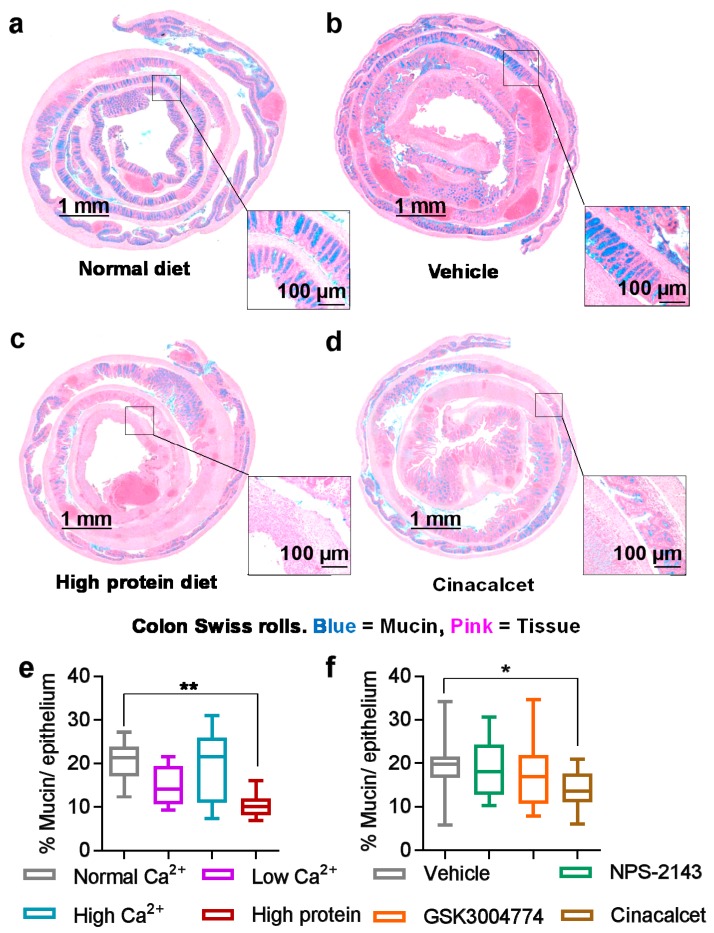
High dietary protein or treatment with cinacalcet reduced mucin abundance in colitic mice. Exemplary images of colon Swiss rolls from colitic mice fed with a normal diet (**a**), treated with vehicle (**b**), fed with a high-protein diet (**c**) or treated or cinacalcet (**d**) and stained with alcian blue for mucin. Quantification of mucin per epithelium from mice fed with experimental diets (**e**) or treated with CaSR allosteric modulators (**f**). Images were linearly contrast and brightness enhanced here for visual clarity only. Statistical significance was determined by one-way ANOVA. * *p* < 0.05, ** *p* < 0.01 and *** *p* < 0.001 were regarded as statistically significant. Data are presented as the mean ± SEM, *n* = 10 and 25 mice per group, for the diets and modulators experiments, respectively.

Mucin plays a major role in the front line of defense as part of the mucous layer in the GI tract [32]. Therefore, we assessed mucin content in the colon as a correlate for chemical barrier integrity and recovery of crypt architecture. The inflammatory damage caused by the high protein diet coincided with a significant reduction in mucin (Figure 4c) compared with the normal diet (Figure 4a), probably due to the destruction of the epithelial layer. The nutritional agonist Ca^2+^ had no effect on mucin content (Figure 4e), while the positive allosteric modulator cinacalcet caused a small yet statistically significant reduction in mucin content (Figure 4d) compared with the vehicle control (Figure 4b). Neither NPS-2143 nor GSK3004774 affected mucin content (Figure 4f).

### 3.4. Inflammatory Cytokines are Differentially Affected by Nutritional and Pharmacological CaSR Ligands

Inflammatory cytokines are quantitative markers of inflammation in DSS models [33]. We measured a panel of cytokines in the plasma to examine the effects of the diets and allosteric modulators and to determine the extent of the systemic inflammation. Indeed, the high protein diet caused a significant increase in the expression of pro-inflammatory markers in the plasma, compared with the normal diet, such as TNF-α (5.0-fold, *p* < 0.05) and IL-1α (13.4-fold, *p* < 0.05) (Table S2). Similarly, cinacalcet significantly increased the expression of TNF-α (4.4-fold, *p* < 0.05), IL-6 (4.9-fold, *p* < 0.05) and IL-1α (2.3-fold, *p* < 0.05) (Table S2).

**Figure 5:**
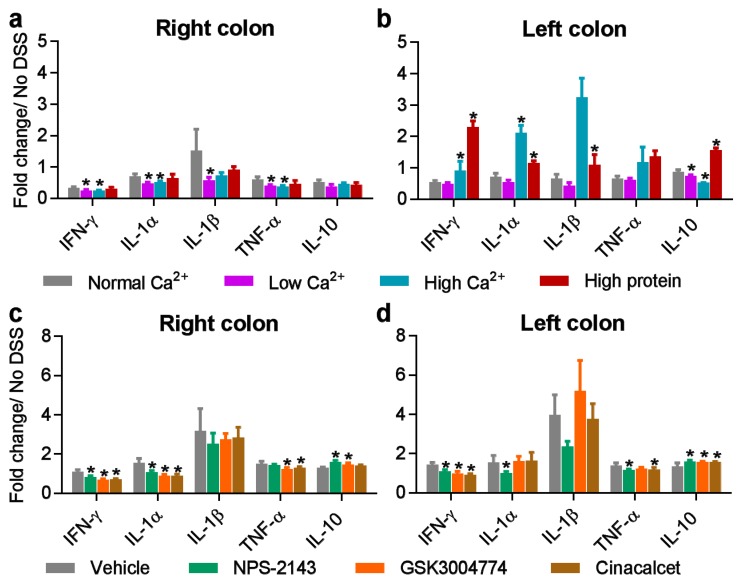
The effects of dietary calcium, dietary protein and CaSR allosteric modulators on inflammatory cytokines in the colons of colitic mice. Cytokine expression in the left (**a**) and the right (**b**) colons of colitic mice fed with different diets. Cytokine expression in the right (**c**) and the left (**d**) colons of colitic mice treated with CaSR allosteric modulators. Fold change is relative to no-DSS controls. Statistical significance was determined by one-way ANOVA. * *p* < 0.05, ** *p* < 0.01, *** *p* < 0.001 were regarded as statistically significant. Data are presented as the mean ± SEM. Samples were pooled with a minimum of 2 per pool and measured in duplicates. No DSS controls, *n* = 4. Experimental diet groups, *n* = 3. Allosteric modulator groups, *n* = 6.

Next, we examined the effects of dietary calcium and protein, as well as the CaSR allosteric modulators, on the expression of cytokines in lysates from the right and left colons. In the right colon, both low and high levels of dietary calcium reduced the expression level of many cytokines, including IL-1β and IFN-α (Figure 5a). High dietary protein caused an upregulation of Gro-α/KC, MIP1- and IL-17, while it caused a downregulation of IL-22 (Table S3). In the left colon, we observed a robust upregulation of most cytokines by DSS (Table S4). Both high dietary calcium and protein increased the expression of several cytokines (Table S4). These included IL-1β and TNF-α (Figure 5b). Interestingly, high dietary calcium decreased the expression of the anti-inflammatory cytokine, IL-10, compared with the normal diet (Figure 5b). By contrast, high dietary protein caused a marked increase in IL-10, compared with the normal diet (Figure 5b).

The allosteric modulators of the CaSR differentially affected the levels of cytokines in the right and left colons (Tables S3 and S4). In the right colon, all three modulators increased the expression of IFN-α (Table S3), whereas NPS-2143 and GSK3004774 increased the expression of IL-10 (Figure 5c). Moreover, cinacalcet and GSK3004774 reduced the expression of TNF-α and IL-1 α (Figure 5c). In the left colon, all three modulators decreased the expression of IFN-α, GM-CSF and IFN-γ (Table S4) but increased the expression of IL-10 (Figure 5d).

### 3.5. NPS-2143 Reduced Immune Cell Infiltration into the Colon

**Figure 6:**
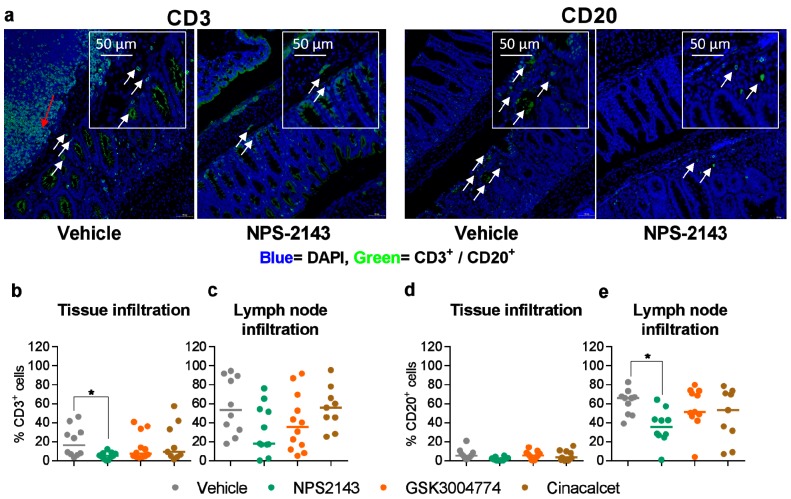
NPS-2143 reduces immune cell infiltration into the colon. (**a**) Representative images of colon sections stained with DAPI (blue) and cell markers for CD3 or CD20 (green) from colitic mice treated with vehicle or NPS-2143. White arrows indicate tissue infiltration, while red arrows indicate lymph node infiltration. Images were acquired with TissueFAXS and the percentage of CD3+ and CD20+ cells per tissue section was quantified using TissueQuest. CD3+ cell infiltration into tissue (**b**) and lymph nodes (**c**) in colitic mice treated with the allosteric modulators of the CaSR. CD20+ cell infiltration into tissue (**d**) and lymph node (**e**) in colitic mice treated with the allosteric modulators of the CaSR. Data are presented as the mean ± SEM. Statistical significance was determined by Kruskal–Wallis test. * *p* < 0.05, ** *p* < 0.01 and *** *p* < 0.001 were regarded as statistically significant. (Vehicle, *n* = 10), (NPS-2143, *n* = 11), (GSK3004774, *n* = 12), (cinacalcet, *n* = 9).

We also compared the extent of the infiltration of CD3^+^ T cells and CD20^+^ B cells into the colon among the different dietary and pharmacological treatments. Dietary calcium and protein did not affect CD20 infiltration (Figure S7). Interestingly, NPS-2143 significantly reduced the infiltration of CD3^+^ and CD20^+^ cells into the colon, compared with the vehicle control (Figure 6a,b,e). Neither GSK3004774 nor cinacalcet affected the infiltration of CD3^+^ or CD20^+^ cells into the colon (Figure 6b–e).

## 4. Discussion

Our findings revealed that dietary calcium and protein differentially affect key parameters in the DSS colitis mouse model. Supplemental calcium was associated with reduced chronic colitis symptoms in DSS-treated mice [34]. However, we found no effects of dietary calcium on acute inflammation in our model, as none of the studied parameters were affected by the calcium content in the diet. It is plausible that the protective effects of calcium, which were reported to influence epithelial barrier integrity, are obsolete in the acute DSS model, where epithelial destruction occurs to a large extent and at a very early stage of the disease. It is noteworthy that basal and inducible levels of most of the analyzed cytokines were significantly higher in the left colon, which is the segment that is mostly examined in DSS models. In our study, dietary calcium increased the expression of several cytokines in the left colon, e.g., IL-1β. This was perhaps due to the chemoattractant effect of calcium on monocytes [35].

Amino acids and peptides resulting from protein breakdown in the gut may act as agonists for the luminal CaSR in the intestines [24] and thereby influence disease activity. In our model, however, we found that high dietary protein exacerbated colitis symptoms, which agrees with previous studies [36,37,38]. This confirms that a high protein diet, while potentially modulating the CaSR, has a deleterious effect on colitis on its own. In light of the results with the positive pharmacological modulators, it seems implausible that these effects of the high protein diet are actually CaSR related. The exact mechanism by which high dietary protein exerts such negative effects is unknown. Several studies showed that dietary content affects microbiota composition, which in turn influences the severity of inflammation [38]. Amino acid-derived metabolites, such as ammonia, p-cresol and hydrogen sulfide were shown to impact the epithelium negatively and increase intestinal permeability [37]. Additionally, it was reported that extraintestinal mechanisms might be involved in DSS colitis models. For instance, excess nitrogenous supply to the kidneys may worsen the systemic inflammation by impairing renal functions [39]. Moreover, DSS-induced liver inflammation was influenced by dietary parameters [40]. Lan et al. showed that high protein content facilitated recovery from colitis in the resolution phase [36]. However, we did not detect any improvement in the clinical score during the short 3-day resolution phase.

Our results also suggest that the allosteric modulators of the CaSR differentially influence key parameters in the DSS-induced colitis mouse model. Contrary to previous reports [19,23], we found that the negative allosteric modulator NPS-2143 ameliorated the clinical symptoms of colitis leading to a lower clinical score compared with vehicle and reduced the infiltration of immune cells. The anti-inflammatory effects of NPS-2143 were already described in lung tissue, where it alleviated allergen-induced asthma and airway hyperresponsiveness in rodents [16]. The observed anti-inflammatory effects of NPS-2143 may be mediated partly by inhibition of the CaSR in circulating immune cells [41,42]. It is also plausible that NPS-2143 may have dual effects depending on the route of administration. Our study is the first to use oral administration of CaSR allosteric modulators in pre-clinical colitis models.

The positive allosteric modulators cinacalcet and GSK3004774 had no effect on clinical score, colon length or histology score. We only detected a reduction in mucin abundance in the mice treated with cinacalcet. Concomitantly, pro-inflammatory markers in the plasma, such as, TNF-α, IL-6 and IL1α were increased by cinacalcet, but not by the gut-restricted GSK3004774. This suggests a systemic effect of cinacalcet, which was lacking in the non-absorbable calcimimetic GSK3004774. Conversely, in the colons, both calcimimetics decreased the expression of pro-inflammatory markers such as IL-1α and IFN-γ. This may be explained by the tissue-specific effects of cinacalcet. In the plasma, CaSR activation in circulating monocytes was associated with activation of the inflammasome [42]. However, these effects may differ in the local environment of the gut. None of the diets or the allosteric modulators of the CaSR affected proliferation or epithelial integrity of colonocytes, as shown in the Ki67 and β-catenin levels (Figure S6a–d). As opposed to cinacalcet, GSK3004774, which would only act on the apical CaSR in the colonocytes, did not affect mucin abundance, suggesting that the deleterious effects of cinacalcet may be mediated systemically, e.g., through the activation of the basolateral intestinal CaSR that is exposed to circulating drugs. To date, there are no pre-clinical reports on the effects of cinacalcet, or other CaSR positive allosteric modulators, on intestinal inflammation. Nonetheless, one of the adverse side effects of cinacalcet is upper GI bleeding. Although the underlying mechanism is unknown, it is possible that this arises as a result of CaSR activation in gastric cells [43]. Our extensive cytokine analysis revealed higher basal and DSS-inducible cytokine expression in the left colon compared with the right colon. The allosteric modulators differentially affected the expression of several cytokines. However, most of the effects, such as the downregulation of IFN-α and IFN-γ, do not support CaSR dependence, as the effect was caused by both the agonist, cinacalcet, and the antagonist, NPS-2143. It is thus possible that these result from off-target effects.

Taken all the parameters into consideration, our data suggests that inhibiting the CaSR with orally administered calcilytics may alleviate colitis symptoms. Moreover, our data show for the first time that orally administered positive allosteric modulators of the CaSR do not alleviate clinical symptoms of colitis. Moreover, cinacalcet reduced mucin abundance and increased TNF-α, IL-6 and IL1α in the plasma, suggesting a systemic pro-inflammatory effect. Our interpretation of these findings considers the limitations of the acute DSS model. First, the short period of resolution may not capture the full extent of recovery. Therefore, our findings need to be extended into the chronic model, with a lower DSS concentration and a longer resolution phase. Second, daily administration of drugs via oral gavage exposes mice to stress and therefore aggravates inflammation. Although necessary for the acute model, it can be replaced by less frequent administration in the chronic model. Finally, the effects of NPS-2143 will require further testing.

## 5. Conclusion

The novel findings of this study show that selective calcimimetics are associated largely with systemic pro-inflammatory effects, without improving the symptoms of DSS-induced colitis. On the other hand, calcilytics may be beneficial in reducing symptoms of colitis. These results highlight unprecedented roles of the CaSR in intestinal inflammation and merit further investigation.

## Figures and Tables

**Table 1:** Protein and calcium content in the standard semi-synthetic diet (LASCRdiet™ LasVendi).

Ingredients	Amount (g/kg Diet)			
	Normal Ca^2+^	Low Ca^2+^	High Ca^2+^	High Protein
Casein (> or =85% protein)	140.000	140.000	140.000	260.000
Supplemental CaCO_3_	12.495	0.000	37.485	12.495

## Supplementary Materials

The following are available online at https://www.mdpi.com/2072-6643/11/12/3072/s1. Figure S1: Intracellular calcium measurements in HEK-CaSR cells. Figure S2: Body weight changes prior to and during colitis induction. Figure S3: Survival analysis. Figure S4: Intact parathyroid hormone (PTH) concentration in the plasma of mice treated with allosteric modulators by gavage. Figure S5: The effects of gavage on clinical score and IL-1α levels in the plasma and the left colon. Figure S6: Ki67 and β-catenin staining in colon sections from DSS-treated mice either fed with experimental diets or treated with allosteric modulators of the CaSR. Figure S7: Immune cell infiltration in the colon in colitic mice fed with the experimental diets. Table S1: Composition of the standard semi-synthetic diet (LASCRdiet™ LasVendi). Table S2: Cytokine expression in the plasma. Table S3: Cytokine expression in the right colon. Table S4: Cytokine expression in the right colon.
