# Supplementary material for: Nutritional and Pharmacological Targeting of the Calcium-Sensing Receptor Influences Chemically Induced Colitis in Mice
Source: Nutrients. 2019 Dec 16;11(12):3072. doi: 10.3390/nu11123072 (PMC6950720; doi:10.3390/nu11123072)
Supplement: Supplementary file 1 [file nutrients-11-03072-s001.zip › Supplementary materials/Supplementary figures.pdf]

Supplementary figures

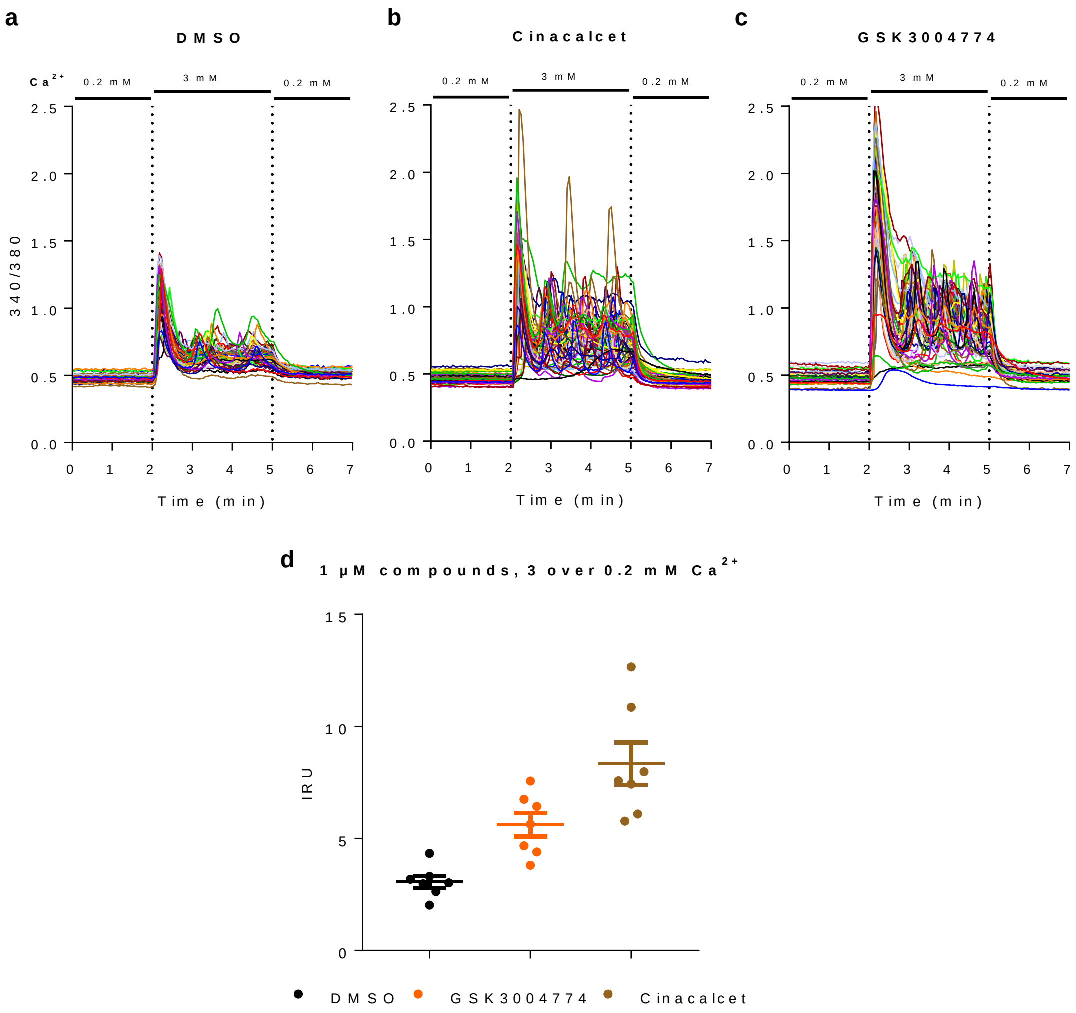

Figure S1. Intracellular calcium measurements in HEK-CaSR cells. The activity of the synthetic calcimimetic (GSK3004774) was validated against cinacalcet by measuring intracellular calcium in HEK-CaSR cells, as described in the methods section. Exemplary traces showing the FURA 340/380 ratio in response to 3 mM Ca^{2+} in the presence of DMSO (a), cinacalcet (b) and GSK3004774 (c). Integrated response units (IRU) in response to 3 mM Ca^{2+} were normalised to basal levels (0.2 mM Ca^{2+}) (d). Data is presented as mean ± SEM, n=7. Statistical significance was determined by one-way ANOVA. *P<0.05 and **P<0.01 were regarded as statistically significant.

Supplementary figures

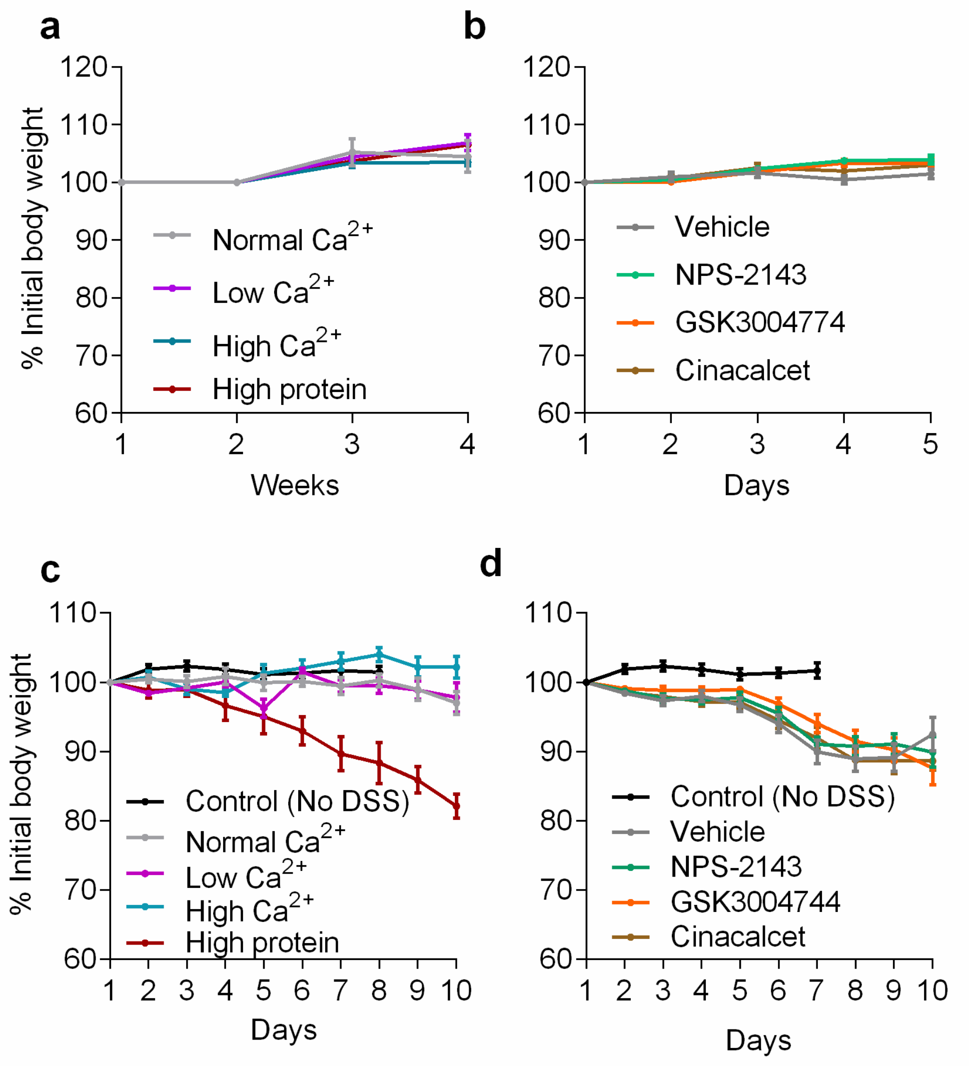

Figure S2. Body weight changes prior-to and during colitis induction. % initial body weight of mice fed with experimental diets 4 weeks prior to DSS administration (a) or treated with CaSR allosteric modulators by gavage (b). % initial body weight changes during DSS administration in mice fed with experimental diets (c) or treated with CaSR allosteric modulators by gavage (d). Mice which received the experimental diets were weighed weekly prior to DSS administration and daily during DSS administration until euthanasia. Mice which received the allosteric modulators by gavage were weighed daily from 5 days prior to DSS administration until euthanasia. Data are presented as mean ± SEM, n= 10 and 25 mice per group, for the diets and modulators experiments, respectively.

Supplementary figures

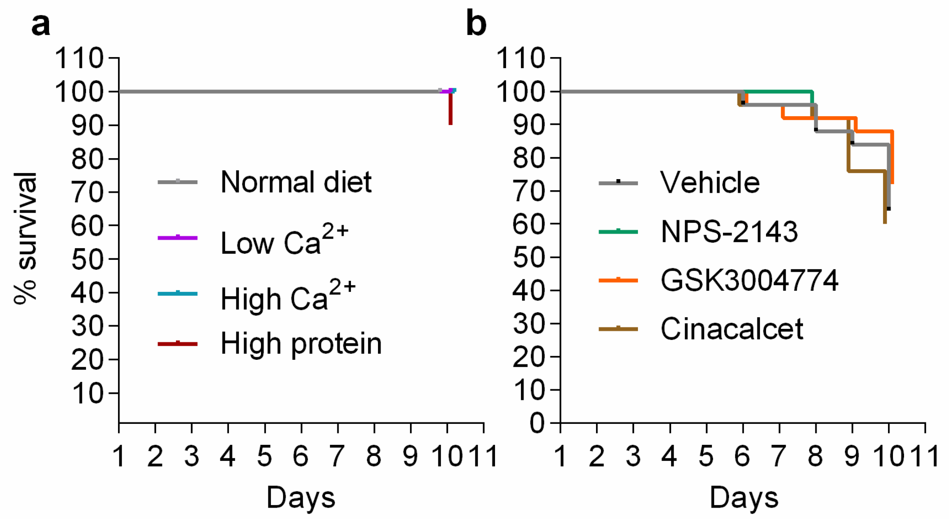

Figure S3. Survival analysis. Survival curves of colitic mice either fed with different diets (a) or treated with CaSR allosteric modulators (b). n= 10 and 25 mice per group, for the diets and modulators experiments, respectively.

Supplementary figures

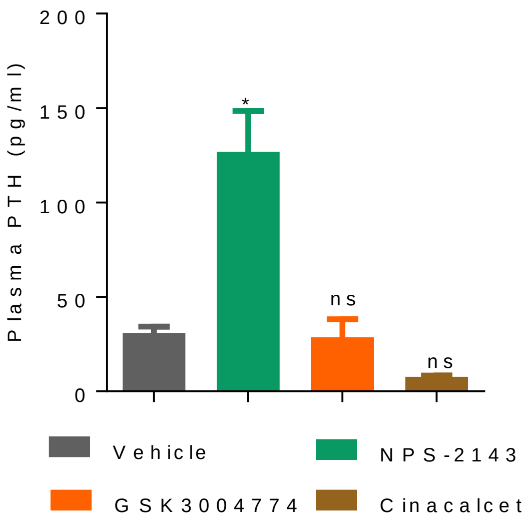

Figure S4. Intact parathyroid hormone (PTH) concentration in the plasma of mice treated with allosteric modulators by gavage. Blood was collected by heart puncture 1 hour after gavage, centrifuged at 2,000 *x* g for 5 minutes and plasma was collected in EDTA-free tubes. PTH measurements were carried out as described in the methods section. Data is presented as mean ± SEM, (Vehicle, n=10), (NPS-2143, n=15), (GSK3004774, n=5) and (Cinacalcet, n=3). Statistical significance was determined by one-way ANOVA. *P<0.05 was regarded as statistically significant.

Supplementary figures

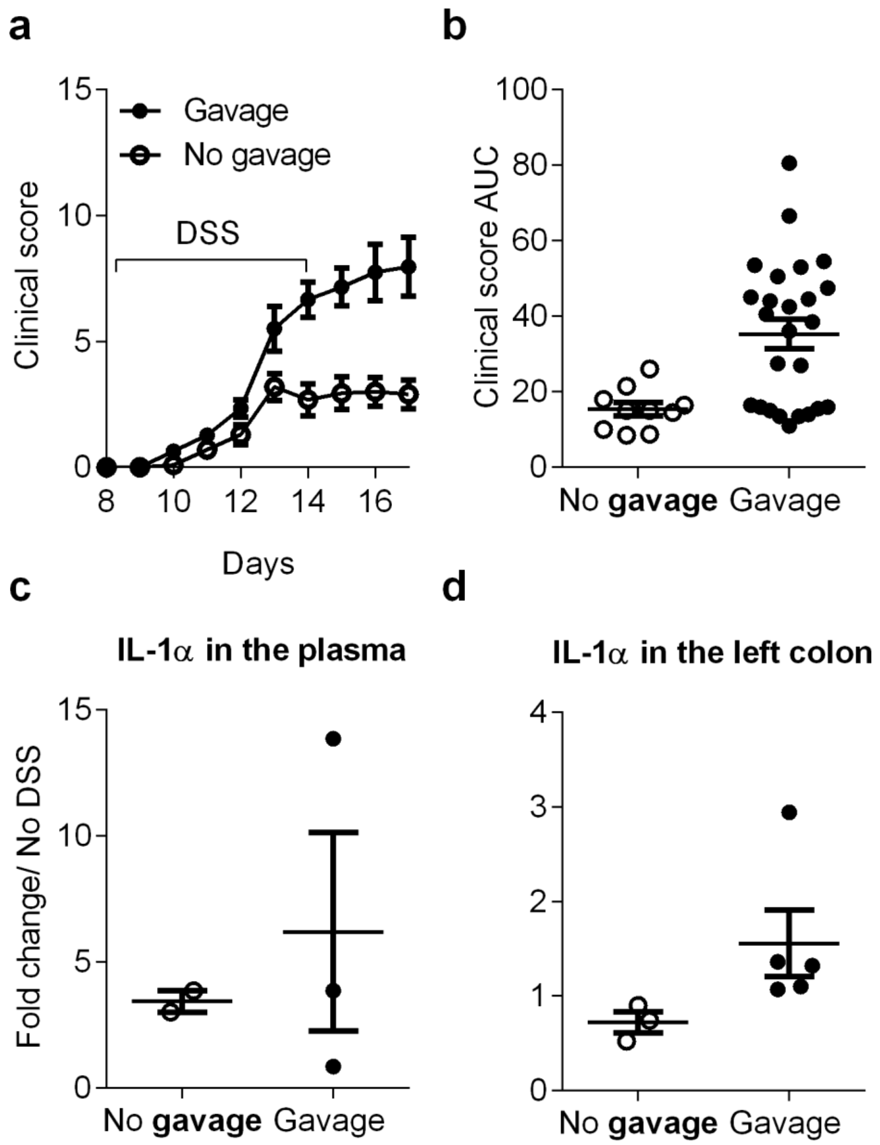

Figure S5. The effects of gavage on clinical score and IL-1α levels in the plasma and the left colon. (a) Clinical score over the colitis induction and resolution phases. (b) Area under the curve (AUC) of the cumulative clinical score, n= 10 and 25 mice per group, for the diets and modulators experiments. IL-1α levels in the plasma (c) and in the left colon (d), samples were pooled with a minimum of 2 per pool, (No gavage, n= 10) and (gavage, n= 16). No gavage (open circles) and gavage-treated groups (closed circles) both received the normal calcium (0.5%) diet. Data are presented as mean ± SEM.

Supplementary figures

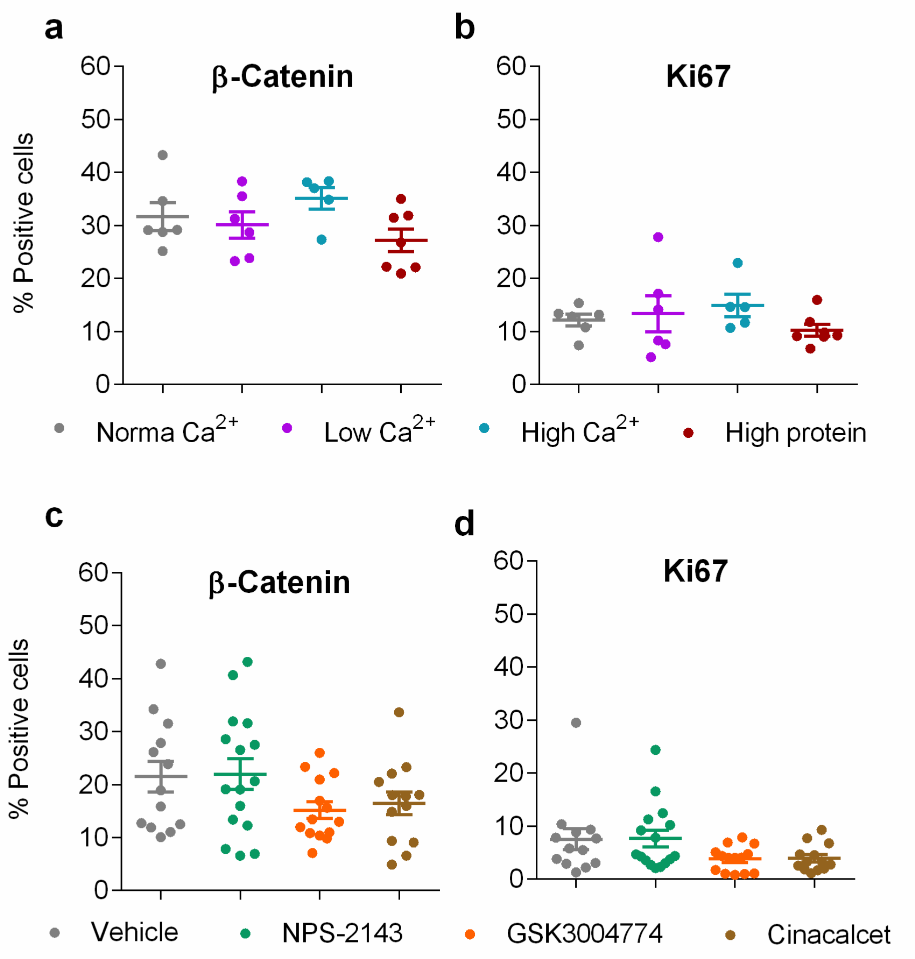

Figure S6. Ki67 and β-Catenin staining in colon sections from DSS-treated mice either fed with experimental diets or treated with allosteric modulators of the CaSR. Paraffin-embedded sections were stained with Ki67 as a proliferation marker and β-Catenin. Images were acquired with TissueFAXS and the percentage of positive cells was quantified with TissueQuest. (a) Percentage of β-Catenin positive cells, and (b) percentage of Ki67positive cells in colitic mice fed with the experimental diets. (c) Percentage of β-Catenin positive cells, and (d) percentage of Ki67positive cells in colitic mice treated with allosteric modulators of the CaSR. Data is presented as mean ± SEM. (Normal and low Ca^{2+}, n=6), (High Ca^{2+}, n= 5), (High protein, n=7). (Vehicle and cinacalcet, n=13), (NPS-2143, n=16), (GSK3004774, n=14).

Supplementary figures

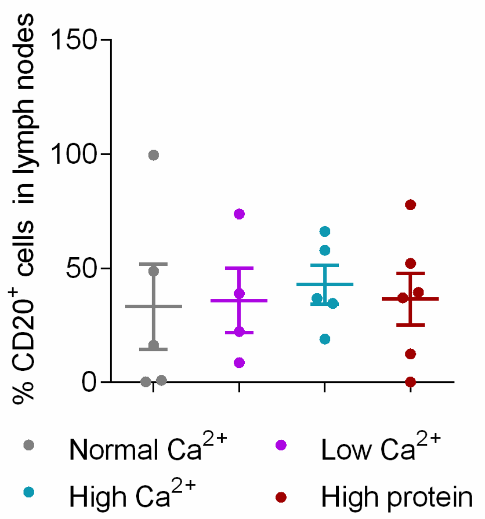

Figure S7. Immune cell infiltration in the colon in colitic mice fed with the experimental diets. Paraffin-embedded colon sections were stained with a CD20 cell marker. Images were acquired with TissueFAXS and the percent of $CD20^{+}$ cells was quantified with TissueQuest. Data is presented as mean ± SEM. (Normal and high Ca^{2+}, n=5), (Low Ca^{2+}, n= 4), (High protein, n=6).
