# Supplementary material for: Nutritional and Pharmacological Targeting of the Calcium-Sensing Receptor Influences Chemically Induced Colitis in Mice
Source: Nutrients. 2019 Dec 16;11(12):3072. doi: 10.3390/nu11123072 (PMC6950720; doi:10.3390/nu11123072)
Supplement: Supplementary file 1 [file nutrients-11-03072-s001.zip › Supplementary materials/Supplementary tables.pdf]

Table S1. Composition of the standard semi-synthetic diet (LASCRdiet™ LasVendi).

Ingredients	Amount (g/kg diet)			
	**Normal Ca^{2+}**	**Low Ca^{2+}**	**High Ca^{2+}**	**High protein**
Corn Starch	465.692	465.692	465.692	465.692
**Casein (> or =85% protein)**	**140.000**	**140.000**	**140.000**	**260.000**
Dextrinized Corn Starch (90-94% tetrasaccharides)	155.000	155.000	155.000	155.000
Sucrose	100.000	100.000	100.000	100.000
Soybean Oil (no additives)	40.000	40.000	40.000	40.000
Fiber	50.000	50.000	50.000	50.000
Mineral Mix (AIN-93M-MX), $CaCO_3$	35.000	35.000	35.000	35.000
**Supplemental $CaCO_3$**	**12.495**	**0.000**	**37.485**	**12.495**
Vitamin Mix (AIN-93-VM)	10.000	10.000	10.000	10.000
L-Cystine	1.800	1.800	1.800	1.800
Choline Bitartrate (41.1% choline)	2.500	2.500	2.500	2.500
Tert-butylhydroquinone	0.008	0.008	0.008	0.008

Table S2. Cytokine expression in the plasma. Cytokine measurements were carried out using the 36-Plex Mouse ProcartaPlex Panel 1A. Blood was obtained by heart puncture in EDTA-free tubes and plasma was collected by centrifugation at 2000 *x* g for 5 minutes. Samples were pooled with a minimum of 2 per pool and measured in duplicates. N.D denotes not detected. For samples with a standard deviation= 0, data was obtained from only one technical duplicate. Data is presented as mean ± SD (pg/ml). Statistical significance was determined by one-way ANOVA with Dunnet's multiple comparisons. P<0.05, P<0.01 and P<0.001 were regarded as statistically significant. *Indicates comparisons to the normal diet control. # indicates comparisons to the vehicle control.

**Cytokine expression in the plasma. Mean ± SD (pg/ml)**									
**A. Th1/Th2 cytokines.**									
	**Control (No DSS) (n= 10)**	**Normal diet (n= 10)**	**Low calcium (n= 10)**	**High calcium (n= 10)**	**High protein (n= 9)**	**Vehicle (n= 16)**	**NPS-2143 (n= 23)**	**GSK3004774 (n= 18)**	**Cinacalcet (n= 15)**
IFN-y	1.9 ± 0.6	2.2 ± 1	3 ± 2.7	3.7 ± 0#	12.7 ± 14.8	1.7 ± 1	1.4 ± 0.8	2.4 ± 2.2	1 ± 0.2
IL-5	9.8 ± 2.1	6.6 ± 0	8.2 ± 0	4.3 ± 0	8.6 ± 6	6.7 ± 4	15.2 ± 12.9*	4.3 ± 0	3.2 ± 1.5
IL-6	24 ± 10.1	42.3 ± 0.5	39.3 ± 6	34.6 ± 0	756.4 ± 1006.1#	32.8 ± 20.3	50.3 ± 45.2	43.6 ± 58.6	160.3 ± 293.3*
IL-12p70	15.3 ± 3.3	12.4 ± 2.9	8.4 ± 2.9#	9.1 ± 0#	12.5 ± 3.3	8.9 ± 5.4	7.2 ± 4.2	8.2 ± 1.4	6 ± 4.2
IL-13	12.1 ± 8.6	3.2 ± 0.2	9.4 ± 2.8#	5.9 ± 0#	4.5 ± 2	4.8 ± 3.4	2.4 ± 3.1*	2.5 ± 2	2.9 ± 1.9
IL-18	203 ± 43	292.2 ± 42.2	240.1 ± 70.6#	N.D	669.3 ± 0	230.4 ± 0	149.9 ± 0	397.7 ± 0	227.3 ± 85.1
TNFα	9.4 ± 2.3	11.3 ± 0.5	11 ± 1.9	10.5 ± 0	47.3 ± 49.3#	9 ± 1.3	10.5 ± 2.1	10.3 ± 8	39.3 ± 60.8*
**B. Th9/Th17/Th22/Treg cytokines.**									
IL-9	162.2 ± 98.3	65.9 ± 14.7	91.6 ± 9.7#	62.4 ± 0	106.3 ± 27#	48.7 ± 18.3	46.1 ± 13.7	54 ± 19.8	53 ± 32.1
IL-22	74.7 ± 0	100.3 ± 36.2	74.7 ± 0	57.3 ± 0	195.6 ± 73.8#	59.1 ± 39.6	65.1 ± 36.8	143.3 ± 0	186.4 ± 198.5*
IL-23	5.8 ± 1.9	5.8 ± 0	8.7 ± 2.3#	3.5 ± 0	6.9 ± 4.8	3.1 ± 0.7	4.6 ± 1.6*	6.2 ± 2.9*	4.1 ± 0.8
IL-27	167 ± 118.1	29.8 ± 23.3	31.3 ± 6.4	35.9 ± 0	58.8 ± 35.8#	17.7 ± 14	30.9 ± 16.9	20.2 ± 17.2	37.6 ± 23.1*
**C. Cytokines and chemokines.**									
Eotaxin	2706.4 ± 165.3	2853.6 ± 471.7	2657.9 ± 30.4	2613.9 ± 0	3141.6 ± 247.7	2707.7 ± 406.8	2459.8 ± 715.4	2610.9 ± 716.8	2971.5 ± 591
GRO-a/kc	13.5 ± 0	27.7 ± 21.2	22.9 ± 1.8	9.4 ± 0	40.9 ± 39.6	76.3 ± 50.9	54.3 ± 33.4	81.7 ± 65	89.1 ± 89.9
IP-10	37.4 ± 5.1	36.1 ± 7.6	47.4 ± 1.9#	32.4 ± 0	48.4 ± 12#	39.5 ± 11.8	36.3 ± 16.4	38.7 ± 13.6	38.4 ± 14.7*
MCP-3	72 ± 4.3	107.4 ± 9.4	112.7 ± 21.4	118.7 ± 0	181.6 ± 103.3#	114 ± 29.2	143 ± 38	95.7 ± 45.1	210.1 ± 192.9
MIP-1a	3.6 ± 1.8	1.6 ± 0.2	1.8 ± 0.4	1.6 ± 0	3.3 ± 0.9#	1.5 ± 0.3	1.8 ± 0.6	2.2 ± 1	2.6 ± 1.5
RANTES	46 ± 12.1	41 ± 4.6	46.6 ± 17.4	35.9 ± 0	45.1 ± 4.2	30.4 ± 6.8	26.9 ± 9.3	27 ± 10.7	34.6 ± 12.1
MIP-1b	2.4 ± 0.4	2.1 ± 0.1	2.3 ± 0.2#	2 ± 0	3 ± 0.1#	2.1 ± 0.2	2.4 ± 0.4	2 ± 0.3	2.6 ± 0.7*
MIP-2	3.6 ± 0.6	2.5 ± 0	4 ± 0.7	2.7 ± 0	7.8 ± 6.4#	3.4 ± 0.4	3.2 ± 0.8	4.7 ± 1.3	8 ± 5*
G-CSF/CSF-3	1.9 ± 0.5	23.7 ± 10.5	9.8 ± 0.1	11.5 ± 0	260.2 ± 258#	54 ± 78.6	33.7 ± 8.2	39.1 ± 48.6	280.7 ± 361*
IL-1α	1.6 ± 0.3	5.5 ± 0.9	4.2 ± 0.7	2.2 ± 0	21.4±13.6#	9.9 ± 10.9	6.2 ± 0.8	6.7 ± 6.6	23 ± 17.9*

Table S3. **Cytokine expression in the right colon**. Cytokine measurements were carried out using the 36-Plex Mouse ProcartaPlex Panel 1A. PBS-flushed colons were separated into right and left sections and lysed in procartaplex buffer. Lysates were then quantified by the BCA method, pooled with a minimum of 2 per pool and measured in duplicates (250 µg/ pool). Data is presented as mean ± SD (pg/ml). Statistical significance was determined by one-way ANOVA with Dunnet's multiple comparisons. P<0.05, P<0.01 and P<0.001 were regarded as statistically significant. *Indicates comparisons to the normal diet control. # indicates comparisons to the vehicle control.

**Cytokine expression in the right colon. Mean ± SD (pg/ml)**									
**A. Th1/Th2 cytokines. Mean ± SD (pg/ml)**									
	**Control (No DSS) (n= 10)**	**Normal diet (n= 10)**	**Low calcium (n= 10)**	**High calcium (n= 10)**	**High protein (n= 9)**	**Vehicle (n= 16)**	**NPS-2143 (n= 23)**	**GSK3004774 (n= 18)**	**Cinacalcet (n= 15)**
GM-CSF	1.3 ± 0.2	1.7 ± 1.2	1.6 ± 0	0.9 ± 0.5	2.4 ± 1.3	6.9 ± 8.1	8.2 ± 2.2	7 ± 1.6	7.3 ± 1.3
IFN-y	7 ± 0.2	2.3 ± 0.4	1.7 ± 0.4#	1.7 ± 0.2#	2 ± 0.6	7.6 ± 1.8	5.7 ± 1.3*	4.7 ± 1.1*	4.9 ± 0.6*
IL-1β	8.6 ± 0.3	13 ± 10.4	4.9 ± 1.5#	6.2 ± 1.3#	7.8 ± 1.5	27.6 ± 23.9	21.7 ± 11.9	23.8 ± 6.1	24.6 ± 11
IL-12p70	4.2 ± 0.7	6.9 ± 1.9	10 ± 6.5	5.6 ± 1.5	4.9 ± 1.3	18.7 ± 8.2	29.1 ± 8.4*	26.2 ± 12.1*	19.8 ± 3.7
IL-13	9.2 ± 0.5	4.4 ± 1	3.4 ± 0.6#	3.3 ± 0.4#	3.9 ± 1.1	12 ± 3.1	12 ± 1.4	10.2 ± 2.1*	10.6 ± 1.1
IL-18	351.9 ± 16.1	351.1 ± 57.7	414 ± 177.1	297.4 ± 20.4	414.9 ± 78.1	3107.2 ± 3209.5	4572.6 ± 1375.5	4853.6 ± 1961.6	11636.1 ± 10844.9*
IL-2	5.3 ± 0.5	0.7 ± 0.6	0.7 ± 0.8	0.4 ± 0.2	0.7 ± 0.9	7.7 ± 3	6.9 ± 0.8	5.8 ± 1.3*	5.8 ± 0.6*
IL-4	4.1 ± 0.4	2.4 ± 0.4	2.3 ± 0.6	2.4 ± 0.1	2.2 ± 0.5	5.5 ± 1.3	6.3 ± 1.1	5.9 ± 1.6	5.9 ± 0.5
IL-5	16.8 ± 0.2	7.4 ± 2.8	5.1 ± 1.9	4.6 ± 0.7#	6 ± 3.1	17.6 ± 6.9	17 ± 2.9	14.1 ± 5.3	12.8 ± 1.8*
IL-6	40.3 ± 4.3	60.2 ± 37.6	38.2 ± 26.4	24.5 ± 7#	72.4 ± 22	1443 ± 3035.3	603.1 ± 1273.5	330.6 ± 286.7	408.6 ± 478.5
TNF- α	56.3 ± 3.5	33.8 ± 9	22.9 ± 3.3#	21.2 ± 3.7#	25.9 ± 10.4	84 ± 19.5	80.5 ± 8	68.9 ± 12.9*	72.8 ± 9.6*
**B. Th9/Th17/Th22/Treg cytokines.**									
IL-10	178.5 ± 8.4	92.7 ± 21.4	68 ± 22#	82.5 ± 10.9	77.6 ± 21.4	232.3 ± 14.9	285.5 ± 36*	261.3 ± 41.5*	250.4 ± 21.8
IL-17A	214.1 ± 15.1	180.7 ± 86.4	121.5 ± 33.5	192.3 ± 65.4	255.2 ± 45.2#	273.8 ± 52.5	340.3 ± 60.7*	301.2 ± 73.8	306.9 ± 55.1
IL-22	11.1 ± 1.7	19.8 ± 15.5	24.8 ± 16.1	17 ± 4.4	10.9 ± 3.7#	66.9 ± 81.9	44.3 ± 6.9	46.4 ± 14.8	47 ± 6.1
IL-23	7.5 ± 3.6	1.8 ± 0.1	1.6 ± 0.1	1.6 ± 0.1	1.7 ± 0.2	12.4 ± 6.6	10.3 ± 1.9*	8.4 ± 1.7*	8.2 ± 2.3*
IL-27	92.1 ± 4.6	32.2 ± 3.7	33 ± 5.6	32.2 ± 2.5	30.9 ± 3.6	141.8 ± 4.7	142.7 ± 13.8	140.4 ± 22.2	136.1 ± 8.2
IL-9	317.2 ± 12.4	173.4 ± 47.3	135.3 ± 18.8	148.6 ± 26.2	168.4 ± 53.4	406.7 ± 112.9	432.8 ± 73.9	378.1 ± 97.1	384.8 ± 33.6
**C. Chemokines.**									

Supplementary tables

ENA-78	171 ± 23.3	187.6 ± 79.7	77.6 ± 32.8[#]	80.5 ± 27.1[#]	207.5 ± 79.8	331.3 ± 106.7	256.3 ± 79.5*	228.3 ± 46.8*	216 ± 49.1*
G-CSF/CSF-3	7.6 ± 0.7	3.7 ± 1.3	2.6 ± 0.8	2.3 ± 0	4.3 ± 1	208.5 ± 425.2	246.1 ± 545	64.4 ± 56.5	145.4 ± 151.3
IFN- α	227.8 ± 67.4	72.9 ± 28.1	39.4 ± 26.1[#]	N.D	25.7 ± 0	987.1 ± 548.9	1534.9 ± 74.1*	1523.8 ± 30.8*	1523.9 ± 63.6*
IL-1α	17.2 ± 0.4	11.9 ± 2.6	8.2 ± 1.2[#]	8.8 ± 1.2[#]	11.2 ± 3.8	26.7 ± 9.6	18.4 ± 3.8*	15.5 ± 2.8*	15.4 ± 3.6*
IL-15/IL-15R	12.2 ± 1.4	3.6 ± 0.7	2.8 ± 0.4	2.9 ± 0.5	3.4 ± 1.1	24.7 ± 7.8	28.3 ± 3.6	27.9 ± 3.7	26.5 ± 3
IL-28	9896.7 ± 502.1	5744.1 ± 1264.7	4677.6 ± 743[#]	5844.7 ± 415.4	4938.9 ± 826.7	12870.9 ± 1721.7	12881.9 ± 850.1	11898.9 ± 1506.6	12022.6 ± 1301.1
IL-3	0.5 ± 0	0.2 ± 0	0.2 ± 0	0.2 ± 0	0.2 ± 0	0.8 ± 0	1.1 ± 0.2*	1 ± 0.2	1 ± 0.1*
IL-31	127.5 ± 5.9	45.3 ± 12.3	34.3 ± 3.7[#]	38.8 ± 5.2	40.5 ± 12.1	142.1 ± 44.8	169 ± 53.5	133 ± 58.1	138.9 ± 26.8
LIF	45.9 ± 4.8	36.4 ± 13	30.7 ± 6.7	30.5 ± 4.8	42.9 ± 7	442.3 ± 870.2	208 ± 316.7	115.7 ± 44	182.4 ± 144.9
M-CSF	4 ± 0.1	1.6 ± 0.3	1.7 ± 0.1	2.2 ± 0.1[#]	1.6 ± 0.1	5.8 ± 1.6	6.1 ± 2.9	4.8 ± 0.8	4.6 ± 0.5
**D. Cytokines.**									
Eotaxin	5742.7 ± 219.2	6003.3 ± 367.4	5526.9 ± 220.8	5777.1 ± 369.2	6484.5 ± 120.8	6047.3 ± 1682.7	7261.6 ± 303.5	7476.6 ± 153.7	7325.7 ± 412.8
GRO-a/kc	97.4 ± 9.8	211.5 ± 65.6[#]	127.6 ± 12.6[#]	128.3 ± 31.8[#]	265.1 ± 53.4	495.9 ± 624.6	457.1 ± 517.6	381.2 ± 95.1	427.3 ± 274.5
IP-10	311.7 ± 57.6	479.7 ± 180	291.6 ± 91.6[#]	477.4 ± 42.1	452.9 ± 175.8	480.7 ± 158.9	433.2 ± 157.7	395.9 ± 109.1	351.4 ± 39.7*
MCP-1	75.9 ± 21.8	435.2 ± 116.9	201 ± 96.6[#]	192.9 ± 35.3[#]	526.1 ± 224.2	2118.9 ± 3349.7	1677.4 ± 2937.5	856.4 ± 197	696.9 ± 448.8
MCP-3	272.6 ± 27.3	353.1 ± 127.4	246.9 ± 41.8[#]	291.5 ± 51	366.6 ± 38.9	416.2 ± 224.7	410.9 ± 64.3	610.2 ± 136.5*	470.8 ± 179.4
MIP-1 α	6.9 ± 0.4	14.7 ± 4.9	10.1 ± 0.9[#]	13.2 ± 1.7	16.4 ± 4.2	69.7 ± 122.1	41.5 ± 41.5	31.4 ± 6.4	28.6 ± 15.2
MIP-1β	6.7 ± 0.1	11.5 ± 1.8	7.9 ± 1.7[#]	9.3 ± 0.3[#]	13.4 ± 2.3[#]	40.5 ± 55.7	18.8 ± 11.2	19.5 ± 10.2	18.2 ± 11.9
MIP-2	8.7 ± 0.6	32.9 ± 34.3	6.9 ± 1.4[#]	7.2 ± 0.8[#]	29.3 ± 15.2	123.4 ± 245	63.8 ± 109.7	34.1 ± 24.5	57.5 ± 65.4
RANTES	832.4 ± 241.5	635.6 ± 192.4	980.6 ± 151.6[#]	1034.5 ± 117.9[#]	785.9 ± 197.6	1850.4 ± 786.4	1477.6 ± 425.3	1562.9 ± 527	1235.5 ± 347.5*

Table S4. **Cytokine expression in the left colon**. Cytokine measurements were carried out using the 36-Plex Mouse ProcartaPlex Panel 1A. PBS-flushed colons were separated into right and left sections and lysed in procartaplex buffer. Lysates were then quantified by the BCA method, pooled with a minimum of 2 per pool and measured in duplicates (250 µg/ pool). Data is presented as mean ± SD (pg/ml). Statistical significance was determined by one-way ANOVA with Dunnet's multiple comparisons. P<0.05, P<0.01 and P<0.001 were regarded as statistically significant. *Indicates comparisons to the normal diet control. #Indicates comparisons to the vehicle control.

**Cytokine expression in the left colon. Mean ± SD (pg/ml)**									
**A. Th1/Th2 cytokines.**									
	**Control (No DSS) (n= 10)**	**Normal diet (n= 10)**	**Low calcium (n= 10)**	**High calcium (n= 10)**	**High protein (n= 9)**	**Vehicle (n= 16)**	**NPS-2143 (n= 23)**	**GSK3004774 (n= 18)**	**Cinacalcet (n= 15)**
GM-CSF	4.9 ± 1.5	3.2 ± 0	N.D	14.7 ± 12.6	4.5 ± 0.9	14.1 ± 7.7	7.4 ± 1	8.5 ± 2	7.5 ± 1.7
IFN-γ	4.9 ± 3.8	2.7 ± 0.3	2.4 ± 0.4	4.5 ± 2.5#	11.3 ± 1.6#	7 ± 1.4	5.4 ± 0.9*	4.8 ± 1.3*	4.5 ± 0.7*
IL-1β	13.1 ± 2.7	8.6 ± 3	5.5 ± 2.4	42.6 ± 13.7#	14.4 ± 7.3	52.1 ± 29.9	30.9 ± 8.5	68 ± 50	49.3 ± 24.9
IL-12p70	7.6 ± 1.3	10.1 ± 3.1	5.1 ± 0.5	31.8 ± 38.4	39.8 ± 21.4#	59.6 ± 28	34.3 ± 12.1*	22.7 ± 4*	19.3 ± 4.1*
IL-13	7.7 ± 5.2	4.9 ± 0.7	4 ± 0.9	5.8 ± 2.8	12.2 ± 2.1#	9.2 ± 0.8	8.1 ± 0.5*	8.3 ± 0.6*	7.9 ± 0.8*
IL-18	420.6 ± 33.7	353.6 ± 52.7	411.9 ± 172.7	755.2 ± 578.5#	2499.2 ± 289.1#	1303.8 ± 788.8	1260 ± 740.2	782 ± 313.7	1056.2 ± 784.8
IL-2	4.7 ± 4.5	1.5 ± 0	N.D	1.7 ± 0.6	7.1 ± 1.8	4.8 ± 1.2	4.3 ± 0.9	4 ± 0.7*	3.7 ± 0.6*
IL-4	4 ± 0.6	3.9 ± 1.3	3.2 ± 0.6	4.5 ± 1.6	7.7 ± 1#	5.8 ± 0.9	6 ± 1.2	5.6 ± 1.3	5 ± 0.6
IL-5	13 ± 7	9.7 ± 1.5	7.3 ± 1.3	10.3 ± 5.7	21.8 ± 4.6#	12.8 ± 4	9.9 ± 0.6*	8.7 ± 2.1*	7.2 ± 0.4*
IL-6	135.5 ± 68.2	49.9 ± 27	25.9 ± 11.2	2041.5 ± 3040.8#	118.1 ± 25.4	1011 ± 590.9	406.9 ± 291.3*	824.4 ± 683	675.9 ± 564.2
TNF-α	52.7 ± 26.3	34.7 ± 7.8	32.7 ± 5	62.1 ± 44.6#	71.9 ± 16.9#	73.6 ± 15.8	61.1 ± 7.1*	64.7 ± 9	63.1 ± 12.3*
**B. Th9/Th17/Th22/Treg cytokines.**									
IL-10	146.8 ± 59.3	128.1 ± 17.7	108.7 ± 10#	76.8 ± 3.9#	228.7 ± 19.8#	198.4 ± 57.6	235 ± 23.7	229.4 ± 17.3	229.8 ± 14.2
IL-17A	307.4 ± 129.7	211 ± 75.1	166.7 ± 61.7	863.2 ± 253.5#	336 ± 28.1	366.5 ± 157.8	411.1 ± 76.6	368.3 ± 111.4	349.4 ± 85.2
IL-22	32.1 ± 18.2	16.3 ± 6.8	5.9 ± 2.3	123.3 ± 21.9#	20.1 ± 7.7	79.6 ± 58.9	50 ± 11	59.7 ± 27.9	83.2 ± 62.6
IL-23	3.4 ± 3.1	2 ± 0.1	1.7 ± 0	2 ± 0	8.2 ± 3.1	4.3 ± 0.8	4.5 ± 0.5	5.9 ± 0.9	4.4 ± 0.6
IL-27	63.1 ± 64.5	29 ± 5.2	24.1 ± 2.3	41.5 ± 17.1	99.3 ± 24.6#	92 ± 34.4	107.6 ± 6.1	130.5 ± 34.9*	116.2 ± 10*
IL-9	298.1 ± 137.1	210.3 ± 37.7	183.1 ± 33.2	319.7 ± 65.9	421.8 ± 78.9	323.7 ± 30.2	318.9 ± 24.7	280.1 ± 43.9	267.1 ± 20.7
**C. Chemokines.**									
ENA-78 (LIX)	502 ± 234.6	216.9 ± 103.7	107.1 ± 54.3#	1095.8 ± 143.2#	269.8 ± 41.2	416.5 ± 85.4	471.6 ± 265.8*	421.5 ± 124.8	439.9 ± 196.1

Supplementary tables

G-CSF/CSF-3	9.9 ± 4.6	4.2 ± 1	2.2 ± 0.4	95.1 ± 147.7$^{\#}$	13.3 ± 4.2$^{\#}$	87 ± 132.2	321.7 ± 683.6	109.3 ± 89.2	203.5 ± 195.2
IFN-α	362.6 ± 0	N.D	N.D	N.D	199.5 ± 88.5	1355.9 ± 99.6	1427.6 ± 69.3*	1444.2 ± 72*	1417.5 ± 40*
IL-1α	19.2 ± 4.4	13.8 ± 3.6	10.3 ± 2.3	40.9 ± 7.6$^{\#}$	22.1 ± 2.5$^{\#}$	22.8 ± 2.7	25.4 ± 15.8	31 ± 11.4	31.5 ± 20.2
IL-15/IL-15R	7.6 ± 6.3	4.3 ± 0.4	3.2 ± 0.9	4.3 ± 1	24.7 ± 9.1$^{\#}$	21.4 ± 12.6	28.4 ± 5.5	27.8 ± 3.1	24.8 ± 2.6
IL-28	9275.9 ± 2739.1	7007.6 ± 689.7	6713.5 ± 322.8	7465 ± 1418.1	11562.7 ± 1507.7$^{\#}$	10657.6 ± 1865.8	12187.4 ± 665.5*	10775.8 ± 1757	10387.9 ± 1197.4
IL-3	0.4 ± 0.2	0.2 ± 0	0.2 ± 0	0.2 ± 0	0.6 ± 0.1	0.6 ± 0.2	0.9 ± 0.1	0.9 ± 0.1	0.9 ± 0
IL-31	91.9 ± 61.8	59.2 ± 11.1	46.2 ± 13.3	39.6 ± 2.1$^{\#}$	164.2 ± 24.4$^{\#}$	91.4 ± 23	108.4 ± 17	105.8 ± 32.4	98.8 ± 23.1
LIF	69.3 ± 15.6	49.6 ± 9.8	28.3 ± 10$^{\#}$	621.8 ± 889.5$^{\#}$	91 ± 18$^{\#}$	221.9 ± 134	652.4 ± 1252.3	184.5 ± 81.3	238.7 ± 135.3
M-CSF	3.5 ± 1	3.1 ± 0.7	2.3 ± 0.2	5.3 ± 3$^{\#}$	3.8 ± 0.6	4.7 ± 1.5	8.7 ± 6.3	7.3 ± 2.3	5.6 ± 1
**D. Cytokines.**									
Eotaxin	5619.8 ± 929.3	5375.5 ± 162.7	4054.8 ± 1102.2	7017.4 ± 168.4	6010.1 ± 70.1	7199.5 ± 512.1	7002.9 ± 252.3	7350.2 ± 160.3	7222.6 ± 388.8
Gro-alpha/KC	268.6 ± 120.3	215.5 ± 96.3	104.1 ± 48.6	754.4 ± 408.5$^{\#}$	173.3 ± 17.6	919.2 ± 570.3	461.1 ± 125.1*	665.1 ± 263.9	574.3 ± 242.6*
IP-10	717 ± 400.6	955.4 ± 470	401.6 ± 181.2$^{\#}$	3361.6 ± 2076.8$^{\#}$	289.4 ± 53.2$^{\#}$	1322.8 ± 350	1148.8 ± 422.6	1318 ± 728.3	1057.9 ± 228.5
MCP-1	868.9 ± 589.2	574.2 ± 354.8	151.1 ± 130$^{\#}$	2927.8 ± 420.1$^{\#}$	281.6 ± 237.9	4370.9 ± 2995.6	2044.7 ± 569.5*	2659.8 ± 1196.6*	2354.5 ± 1268.9*
MCP-3	606.8 ± 266.4	458.8 ± 56.4	247.2 ± 103.2$^{\#}$	979.8 ± 70.1$^{\#}$	372.8 ± 113.5	1168.9 ± 202.6	948.1 ± 101.9	1116.3 ± 208.5	1044.7 ± 245.3
MIP-1a	19 ± 9.1	24.3 ± 6.4	10.9 ± 3.7$^{\#}$	54.4 ± 8.9$^{\#}$	13.5 ± 5$^{\#}$	85.3 ± 59.1	66.9 ± 15.5	101.8 ± 30	89.3 ± 47.2
MIP-1b	21.9 ± 12.5	18.6 ± 8.6	9.1 ± 5.1	68 ± 25.8$^{\#}$	10.4 ± 7.4	49.7 ± 21.3	46.5 ± 12.1	57.8 ± 24.5	74.4 ± 51.3
MIP-2	39 ± 26.7	46.9 ± 59.3	9.7 ± 3	197 ± 215.4$^{\#}$	15.5 ± 1.6	194.2 ± 227.7	52.1 ± 26.4*	109 ± 76.6	103.7 ± 91.5
RANTES	907 ± 444.5	680.8 ± 106.3	544.2 ± 310	2118.5 ± 1277.9$^{\#}$	683.6 ± 7.5	1853.4 ± 617.5	2074.2 ± 615.4	2074.4 ± 575.8	1963.8 ± 762.4
